# Genetic population structure of the Xiongnu Empire at imperial and local scales

**DOI:** 10.1126/sciadv.adf3904

**Published:** 2023-04-14

**Authors:** Juhyeon Lee, Bryan K. Miller, Jamsranjav Bayarsaikhan, Erik Johannesson, Alicia Ventresca Miller, Christina Warinner, Choongwon Jeong

**Affiliations:** ^1^ School of Biological Sciences, Seoul National University, Seoul 08826, Republic of Korea.; ^2^ Max Planck Institute for the Science of Human History, Jena 07745, Germany.; ^3^ Museum of Anthropological Archaeology, University of Michigan, Ann Arbor, MI 48109, USA.; ^4^ History of Art, University of Michigan, Ann Arbor, MI 48109, USA.; ^5^ National Museum of Mongolia, Ulaanbaatar, Mongolia.; ^6^ Circle CRM Group Inc., Calgary, AB T2B 2X3, Canada.; ^7^ Department of Anthropology, University of Michigan, Ann Arbor, MI 48109, USA.; ^8^ Max Planck Institute for Evolutionary Anthropology, Leipzig 04103, Germany.; ^9^ Department of Anthropology, Harvard University, Cambridge, MA 02138, USA.

## Abstract

The Xiongnu established the first nomadic imperial power, controlling the Eastern Eurasian steppe from ca. 200 BCE to 100 CE. Recent archaeogenetic studies identified extreme levels of genetic diversity across the empire, corroborating historical records of the Xiongnu Empire being multiethnic. However, it has remained unknown how this diversity was structured at the local community level or by sociopolitical status. To address this, we investigated aristocratic and local elite cemeteries at the western frontier of the empire. Analyzing genome-wide data from 18 individuals, we show that genetic diversity within these communities was comparable to the empire as a whole, and that high diversity was also observed within extended families. Genetic heterogeneity was highest among the lowest-status individuals, implying diverse origins, while higher-status individuals harbored less genetic diversity, suggesting that elite status and power was concentrated within specific subsets of the broader Xiongnu population.

## Teaser

Members of the Xiongnu Empire were genetically diverse, but elite status was concentrated within specific genetic subsets.

## INTRODUCTION

The Xiongnu Empire was the first of many historically documented steppe empires to arise in Eurasia, and its formation foreshadowed the rise of subsequent nomadic imperial powers, including the Mongol Empire, whose reach a millennium later stretched from the East Sea to the Carpathian Mountains (*1*). Centered on the territory of present-day Mongolia, the Xiongnu empire controlled the Eastern Eurasian Steppe and surrounding regions in northern China, southern Siberia, and Central Asia for nearly three centuries, starting from ca. 209 BCE until their eventual disintegration in the late first century CE. At its height, the Xiongnu profoundly influenced the political economies of Central, Inner, and East Asia, becoming a major political rival of imperial China and establishing far-flung trade networks that imported Roman glass, Persian textiles, Egyptian faience, Greek silver, and Chinese bronzes, silks, and lacquerware deep into the heart of their empire (*2*).

The Xiongnu represented a radically new kind of political entity that incorporated heterogeneous nomadic and sedentary groups spanning the Eastern Steppes and as far west as the Altai Mountains, under a single authority. As the Xiongnu expanded their empire from its core in central and eastern Mongolia, they conquered and integrated numerous neighboring groups. They successfully expanded into western Mongolia and southern areas of Lake Baikal, while winning decisive victories in northern China (*3*). However, the Xiongnu were much more than just experts in mobilizing cavalry forces for conquests. They were also shrewd trade partners who exerted considerable influence over the Silk Road kingdoms of Central Asia (*4*), with even greater control over Eurasian exchange networks during the late Xiongnu period (ca. 50 BCE to 100 CE). Nevertheless, a detailed understanding of their internal social and political organization is lacking (*5*).

Historical narratives of the Xiongnu were largely authored by their Han Chinese political rivals, who repeatedly and dismissively characterized their polity as a “simple body” of nomadic elites (*3*, *6*). Much of what is now known about Xiongnu sociopolitical organization has been gleaned from textual evidence alongside a growing body of archaeological sites throughout Inner Asia, consisting primarily of cemeteries (*2*, *7*–*9*). The mortuary record indicates that there is a sociopolitical hierarchy among the Xiongnu, with clear differences between individuals in terms of burial type, investment in construction, and offerings. Most identified graves of the late Xiongnu period are shaft pits set beneath thick stone rings on the surface. These conspicuous burials represent the vast network of regional and local elites of Xiongnu society, while commoners were likely buried under less conspicuous stone piles or in unmarked pits (*10*). The uppermost aristocratic ruling elites of the empire were buried in large square stone tombs, often flanked by satellite burials of lower-status individuals, forming a mortuary complex (*11*). Elites in square tombs and large circular graves were richly buried, typically in decorated wood-plank coffins and accompanied by foreign luxury goods, gold, or gilt objects, and sacrifices of horses and other valuable livestock. Metal discs and crescents representing the sun and moon, a symbol of the Xiongnu empire, are also frequently found in such elite graves. Because of their wealth and conspicuous appearance on the landscape, many Xiongnu graves have been looted since antiquity, but the differences in grave forms nevertheless reflect clear social gradations, with the square tombs as an exclusive political faction within the empire (*2*).

Previous archaeogenetics studies have sought to identify the people who made up the Xiongnu and have found an extremely high level of genetic diversity across the Xiongnu empire (*12*–*16*). Recently, a genome-wide study of 60 individuals from 27 Xiongnu sites found that this diversity was initially formed by the unification of two genetically distinct pastoralist populations in Mongolia—one descending from groups associated with the Deerstone Khirigsuur, Mönkhkhairkhan, and Sagly/Uyuk cultures in the west and the other the descendants of the Ulaanzuukh and Slab Grave cultures in the east—followed by additional population influx from other regions, most likely Sarmatia (near present-day Ukraine) and imperial China (*14*). However, while this evidence supports previous claims that the Xiongnu Empire was likely a multiethnic, multicultural, and multilingual entity, until now, it has not been possible to determine whether such diversity was composed of a heterogeneous patchwork of locally homogenous communities or whether local communities themselves were also internally diverse. Moreover, many aspects of Xiongnu political constituencies still remain unknown, such as who made up the imperial elite occupants of the square tombs, and what their relationship was to lower-status individuals, including those buried in satellite graves within their elaborate tomb complexes. It also remains unclear whether high-status square tomb elites and local elites in the standard circular graves were drawn from the same segments of the Xiongnu population, or whether local elites were more likely to genetically resemble prior local populations than their incoming imperial counterparts, which would suggest that demographic processes associated with empire formation may have been stratified by status and origin.

To address these questions, here, we genetically investigate in detail a range of burials from the aristocratic elite cemetery of Takhiltyn Khotgor (TAK) and the local elite cemetery of Shombuuzyn Belchir (SBB), located at the far western frontier of the empire in Mongolia’s present-day Khovd province. Analyzing the genome-wide data of 18 individuals from high and low-status burials, we show that both communities harbored an extremely high level of genetic diversity that is comparable to that of the Xiongnu Empire as a whole. High genetic diversity is reflected within individual tomb complexes and burial clusters and even extended family groups. Thus, we find that the same sociopolitical processes that produced a genetically diverse empire on a vast scale also operated at the smallest scale, creating highly diverse local communities over the span of only a few generations. There are also discernable genetic patterns with respect to social and political status at TAK and SBB, where individuals of the lowest status (based on grave form and mortuary remains) have the highest degree of genetic heterogeneity. In contrast, higher-status individuals are less genetically diverse and have high levels of eastern Eurasian ancestry. This further suggests the existence of an aristocracy in the Xiongnu empire, that elite status and power was concentrated within specific subsets of the broader population.

## RESULTS

### Generation of genome-wide data from Xiongnu aristocratic elites, local elites, and subordinates

**Fig. 1.**
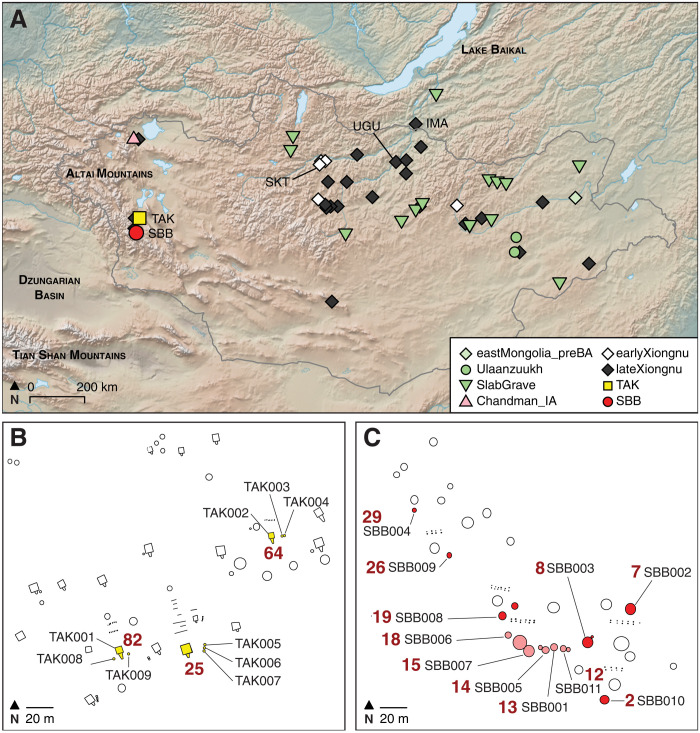
Map of Xiongnu sites in this study, and burial plans of the TAK and SBB cemeteries. (**A**) Geographic locations of the sites analyzed in the study are presented with the cultural affiliation and the time period. Newly sequenced individuals were excavated from the aristocratic elite cemetery of Takhiltyn Khotgor (TAK) (yellow square) and the local elite cemetery of Shombuuzyn Belchir (SBB) (red circle) in western Mongolia. Previously reported Xiongnu sites with five or more sequenced individuals are also labeled in the figure: Salkhityn Am (SKT), Uguumur Uul (UGU), and the Il’movaya Pad (IMA) (*14*). Other Xiongnu sites are indicated with white diamonds (early Xiongnu) or black diamonds (late Xiongnu). Sites of the preceding Early Iron Age (EIA) associated with the Sagly/Uyuk (pink triangle) and Slab Grave (green down-pointing triangle) archaeological cultures are also shown. (**B**) Plan detail of the TAK cemetery indicating the square tomb complexes and associated graves (yellow). See fig. S1 for a full cemetery plan. (**C**) Plan of the SBB cemetery indicating stone circle and stone pile graves. Excavation focused on a dense grave cluster (pink) and a selection of other representative graves (red). See fig. S2 for a full cemetery plan. For both sites, tomb or grave numbers are indicated in bold red; each analyzed individual is numbered in black.

Before this study, two archaeogenetic studies had intensively investigated Xiongnu-era cemeteries in the political core of the Xiongnu empire at Egyin Gol (*12*) and Tamir Ulaan Khoshuu (*16*), but these studies did not generate genome-wide data and thus they have limited capacity to trace individual ancestries and relationships. Other studies have focused on producing genome-wide datasets (*13*–*15*), but the small number of individuals they analyzed per site make these data insufficient to explore genetic diversity within Xiongnu communities or potential associations with sociopolitical status. To address this, we conducted an intensive genome-wide archaeogenetic investigation of two Xiongnu cemeteries, the aristocratic elite cemetery of TAK and the local elite cemetery of SBB, which are located at the far western frontier of the Xiongnu empire in the Altai mountains. These cemeteries include the full social spectrum of individuals from exclusive square tombs to standard circular graves to meager pit graves. This dataset helps to better understand the genetic diversity, heterogeneity, and relationships among elites and subordinates at Xiongnu communities in the social and spatial edges of their empire. We then compared these frontier Xiongnu communities to previously published archaeogenomic data for 29 additional Xiongnu sites across Mongolia (Fig. 1A) (*13*, *14*).

The aristocratic cemetery of TAK, dating to ca. 40 BCE to 50 CE (*17*, *18*), is notable not only for its large circular graves of local elites but also for its numerous square tombs (Fig. 1B and fig. S1), which were reserved for individuals of the highest status within the imperial Xiongnu hierarchy. Flanking many of these tombs are low-status “commoner” graves consisting of simple stone piles over stone cist or earthen pit burials. Together, these square tombs and satellite graves form extended mortuary complexes. At TAK, two complete square tomb mortuary complexes have been excavated, THL-82 and THL-64, and a third complex, THL-25, has been partly excavated (fig. S1). THL-82 consists of a large central elite square tomb flanked by two satellite graves to its east and west (Fig. 1B). The tomb contained the remains of an adult female, TAK001, who was buried in a decorated wood-plank coffin with six horses, Chinese bronze chariot pieces, and a bronze spouted pot (*19*). The use of a wood-plank coffin, in strict adherence with elite Xiongnu political culture and rituals, is particularly noteworthy in this frontier context, as the large larch wood planks must have been imported at great effort and expense into this largely treeless mountain region (*20*). The satellite graves each contained an adult male interred in an earthen pit burial (TAK008 and TAK009), one of whom (TAK008) was interred in a prone (face-down) position, which differs from the supine (face-up) position that is more typical of Xiongnu burials. THL-64 consisted of a large central elite square tomb with two satellite graves on its eastern side (Fig. 1B). Like THL-82, the tomb also contained the remains of an adult female, TAK002, who was buried in a wood-plank coffin with one horse, four caprines (either sheep or goat), and a golden disc and crescent, representing the sun and moon (*17*). The satellite graves each contained an adolescent male (TAK003 and TAK004) buried in simple stone cists in a semi-flexed position, a position consistent with long-standing local mortuary traditions in western Mongolia (*21*). Tomb complex THL-25, for which only the satellite graves have been excavated to date, consisted of a large central square tomb flanked by three satellite graves on its eastern side (Fig. 1B). The three satellite graves consisted of simple earthen pit burials, marked only by small piles of stones, containing the remains of a child (TAK005) and two adult males (TAK006 and TAK007). In total, we genetically investigated eight individuals from TAK cemetery, seven new to this study and one (TAK001) published in a previous study (*14*).

Located approximately 50 km to the southwest of TAK, the local elite cemetery of SBB is situated along a strategic high mountain pass and spans a period from ca. 50 BCE to 210 CE (*18*, *20*). Consistent with other local elite Xiongnu cemeteries, it consists primarily of circular graves containing the remains of both adult females and males, as well as children (data file S1A). Fifteen of the 33 graves have been excavated to date, of which 11 were genetically screened in this study and 10 of 11 were sufficiently preserved for genome-wide analysis (Fig. 1C and fig. S2). The analyzed individuals span the full spectrum of marked social status, from individuals in large stone-encircled graves with decorated wood-plank coffins and elaborate grave goods to humble burials consisting of small stone cists (fig. S2). Five of the analyzed graves were arranged into a cluster (graves 12, 13, 14, 15, and 18), while the others were spatially dispersed and selected as a representative sample of the remainder of the cemetery (graves 2, 7, 8, 19, 26, and 29). Graves 7, 8, 15, and 19 were the highest-status graves analyzed in this study, and each consisted of an adult female buried in a wood-plank coffin surrounded by a stone ring. Grave 7 contained the remains of an older adult female (SBB002) buried with a disassembled wooden cart, a bronze cauldron, a ceramic cooking pot, and a golden sun disc and moon crescent nailed to the wood-plank coffin. Grave 8 contained the remains of an older adult female (SBB003) buried in a quatrefoil-decorated coffin and interred with gilded glass beads and a Chinese mirror fragment, as well as a large deposit of livestock offerings consisting of at least 12 caprines (sheep or goats). Grave 15 contained the remains of an adult female (SBB007) buried in a decorated wood-plank coffin overlain with wooden cart pieces, as well as horse-riding tack, a gilded iron belt clasp, and a Han Dynasty–painted lacquer cup. Grave 19 contained the remains of a young adult female (SBB008) who had apparently died in childbirth; she was buried alongside an infant and wore a paste-bead necklace containing a faience bead depicting the phallus of Bes, an Egyptian god associated with the protection of children. Like SBB003, this woman was also buried with Chinese mirror fragments. The remaining graves were simpler, consisting of a small stone circle or stone pile overlying a stone cist. Grave 13 contained the remains of a middle-aged adult male (SBB001) buried with a bow, arrows, and spear. An adolescent (SBB011) buried in grave 12 was also buried with a bow, arrows, and spear, and a child (SBB009) buried in grave 26 was buried with a child-sized bow. Three additional children were buried in graves 14 (SBB005), 18 (SBB006), and 29 (SBB004), and grave goods consisted of varied glass beads in graves 14 and 18, while the child in grave 29 was buried with silk, leather, and felt. Last, grave 2 (SBB010) contained the remains of an older adult male buried with an iron sun disc and moon crescent. Screening of burial sediments recovered traces of silk clothing in all SBB burials.

For this study, we generated new genome-wide data for 19 individuals from TAK and SBB, of which 17 yielded sufficient human DNA for analysis (>0.1% human DNA), and we further enriched these DNA libraries for a panel (“1240K”) of 1,233,013 ancestry-informative single-nucleotide polymorphisms (SNPs) using an in-solution DNA capture method (*22*). After enrichment and sequencing, between 11,950 and 659,982 SNPs were successfully covered by at least one high-quality read for each individual (data file S1A). For six individuals with endogenous DNA preservation in excess of 30% (SBB003, SBB007, SBB010, TAK002, TAK006, and TAK008), we also produced whole-genome shotgun sequencing data to 0.7 to 2.5× coverage (data file S1A). All libraries exhibited characteristic patterns of ancient DNA damage, including short fragment lengths and cytosine deamination at fragment ends. Genetic sex was determined for all 17 individuals (data file S1A), and all individuals exhibited low contamination (<6%; data file S1A), as estimated using mitochondrial DNA for all individuals (*23*) and the X chromosome for 10 males (*24*). For downstream population genetic analyses, we performed pseudo-haploid genotype calling (https://github.com/stschiff/sequenceTools; v1.5.2 last accessed at 25 April 2022) and concatenated our new genotype data with previously published genotype data for TAK001 (*14*) and other ancient (*13*–*15*, *22*, *25*–*58*) and present-day (*25*, *29*, *59*–*63*) individuals (data file S1B). We also attempted to assign uniparental haplogroups to each individual and successfully determined the mitochondrial haplogroup for 17 individuals and the Y-chromosome haplogroup for 6 of 10 males (data file S1A). We estimated genetic relatedness among individuals from TAK, SBB, and previously reported sites (*14*). Two pairs of genetic relatives were identified at the SBB site: one pair of second-degree relatives (SBB005-SBB007) and a pair of second-degree or more distant relatives (SBB001-SBB005; data file S1E).

### Modeling Xiongnu ancestry

Before proceeding to a more granular genetic analysis of the TAK and SBB sites, and of the Xiongnu population more generally, we first refined and updated our modeling of Xiongnu ancestry to incorporate newly available genome-wide data from the preceding Late Bronze Age (LBA) and Early Iron Age (EIA) periods in central and eastern Mongolia (*15*). In a previous study, we modeled individuals of the early Xiongnu period as a mixture of two distinct genetic groups present in Mongolia during the EIA (ca. 900 to 300 BCE): “Chandman_IA” and “SlabGrave.” Chandman_IA was representative of people in far western Mongolia associated with Sagly/Uyuk (ca. 500 to 200 BCE), Saka (ca. 900 to 200 BCE), and Pazyryk (ca. 500 to 200 BCE) groups in Siberia and Kazakhstan. “SlabGrave” was representative of people in eastern and central Mongolia associated with Slab Grave (ca. 1000 to 300 BCE) mortuary sites (*14*). Likely arising out of the LBA Ulaanzuukh archaeological culture (ca. 1450 to 1150 BCE) in eastern Mongolia, Slab Grave groups expanded into central and northern Mongolia as far north as the Lake Baikal region (*7*, *14*, *64*). Overall, individuals from the Ulaanzuukh and the Slab Grave cultures present a homogeneous genetic profile that has deep roots in the region and is referred to as Ancient Northeast Asian (ANA) (*14*). The recent publication of additional genome-wide data for Ulaanzuukh and Slab Grave individuals (*15*) provided an opportunity to investigate the genetic profile of the Slab Grave individuals across a wider geographical distribution (Fig. 1A) and to refine our genetic modeling of the formation of the Xiongnu more generally. We updated our admixture modeling of Ulaanzuukh and Slab Grave individuals using the qpAdm program (*25*).

**Fig. 2.**
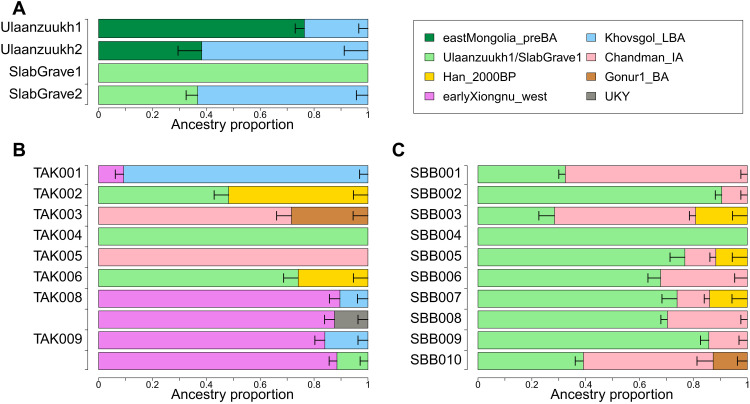
qpAdm modeling of TAK and SBB individuals. The genetic profiles of ancient individuals are modeled as the mixture of two or three populations. The ancestry proportion of each source population is represented by the size of the box on the *x* axis. Horizontal bars represent ±1 SE estimated by qpAdm using 5-cM block jackknifing. Detailed results are presented in data file S2. (**A**) Ulaanzuukh and SlabGrave individuals are modeled as the mixture between Ancient Northeast Asian (ANA), represented by eastMongolia_preBA in this study, and Khovsgol_LBA. We modeled Takhiltyn Khotgor (TAK) (**B**) and Shombuuzyn Belchir (SBB) (**C**) individuals as the mixture of the preceding groups from Mongolia and the surrounding regions: SlabGrave1, Chandman_IA, Han_2000BP, Gonur1_BA, earlyXiongnu_west, Khovsgol_LBA, and UKY. We present two models for TAK008 and TAK009.

First, we detected a subtle genetic shift in eastern Mongolia between the preceding pre–Bronze Age period and LBA Ulaanzuukh individuals (*n* = 13) (Fig. 2A, fig. S3; and data file S2A). We model this difference as gene flow from a nearby LBA population in northern Mongolia, such as that found in the northernmost province of Khovsgol (“Khovsgol_LBA”). Collectively, Ulaanzuukh individuals are adequately modeled as having a 24.5% contribution from Khovsgol_LBA (*P* = 0.550; data file S2A), and at an individual level most also fit the same model with 13.9 to 33.4% contributions from Khovsgol_LBA (data file S2A), with the exception of one individual with an unusually high Khovsgol_LBA contribution (ULN005; 63.5%). On the basis of the admixture modeling results, we grouped 12 of 13 Ulaanzuukh individuals as an analysis unit (“Ulaanzuukh1”), excluding ULN005 for its much higher Khovsgol_LBA affinity (separately analyzed as “Ulaanzuukh2”), and used it as a representative of the Ulaanzuukh gene pool.

To better understand the genetic make-up of Iron Age Slab Grave individuals (*n* = 16), we compared their genetic profiles with the preceding Ulaanzuukh individuals. Incorporating data from 11 newly published individuals (*15*), we identified a subtle genetic heterogeneity not detected in our previous study (*14*). While 13 of 16 individuals are cladal with Ulaanzuukh1, the remaining 3 individuals require additional Khovsgol_LBA ancestry (Fig. 2, fig. S3, and data file S2B). In particular, Slab Grave individuals I6359, I6369, and DAR001 are markedly distinct from the others by carrying high proportions of Khovsgol_LBA ancestry (42.6 to 79.7%) (data file S2B). This pattern, in which most Slab Grave individuals are genetically homogeneous while some have a large and heterogeneous ancestry fraction deriving from a Khovsgol_LBA-like gene pool, is likely due to population mixing in their recent past and is consistent with archaeological evidence that the Slab Grave culture expanded into central and northern Mongolia and replaced the preceding inhabitants in the region with a low level of mixing (*65*). On the basis of individual ancestry modeling, we assigned most of the Slab Grave individuals (13 of 16) into a single group “SlabGrave1,” while we assigned the remaining three individuals with high Khovsgol_LBA ancestry into another group, “SlabGrave2,” for their use in the downstream group-based analyses.

To characterize the genetic profiles of our new Xiongnu-period individuals, we modeled the ancestry composition of the TAK and SBB individuals using qpAdm (Fig. 2). Most individuals (15 of 18) are adequately modeled by the admixture models previously applied to Xiongnu individuals, which used Ulaanzuukh/SlabGrave and Han_2000BP as eastern Eurasian sources (data file S2C) (*14*). Eight of these 15 individuals are adequately modeled with two ancestries, SlabGrave1 and Chandman_IA: Five are mixed between SlabGrave1 and Chandman_IA (SBB001, SBB002, SBB006, SBB008, and SBB009; 32 to 91% from SlabGrave1), one is indistinguishable from Chandman_IA (TAK005), and two are indistinguishable from SlabGrave1 (SBB004 and TAK004). A further five individuals are modeled using an additional East Asian ancestry distinct from ANA, here represented by Han_2000BP (*14*): Three are modeled as a mixture of SlabGrave1, Chandman_IA, and Han_2000BP (SBB003, SBB005, and SBB007; 28 to 77% from SlabGrave1, 11 to 52% from Chandman_IA, and 12 to 19% from Han_2000BP), and two are modeled as a mixture of SlabGrave1 and Han_2000BP (TAK002 and TAK006; 48 to 74% from SlabGrave1 and 26 to 52% from Han_2000BP). Last, two individuals require an Iranian/Central Asian ancestry represented by Gonur1_BA (*26*): One is modeled as the mixture of SlabGrave1, Chandman_IA, and Gonur1_BA (SBB010; 39% from SlabGrave1, 48% from Chandman_IA, and 13% from Gonur1_BA), and the other as the mixture of Chandman_IA and Gonur1_BA (TAK003; 28% from Gonur1_BA). TAK003 has a higher Gonur1_BA-related ancestry proportion than previously described early Xiongnu individuals with the same ancestry combination (“earlyXiongnu_west”), corroborating a previous report of continued gene flow from Central Asia between the early and late Xiongnu periods (*14*).

We note that all qpAdm admixture models equally fit when SlabGrave1 was replaced by “AR_Xianbei_IA” from the Mogushan archaeological site in Inner Mongolia that belongs to the Iron Age Xianbei context (data file S2C) (*27*). All but two males (BUL002 and I6365) associated with the Ulaanzuukh and Slab Grave cultures belong to Y-haplogroup Q, all three AR_Xianbei_IA males belong to Y-haplogroup C, and the Xiongnu males harbor both Q and C (data file S1C) (*14*, *15*). Although not conclusive, this suggests that the ANA ancestry source of the Xiongnu-period individuals may not be exclusively traced back to the Slab Grave culture but may also include nearby groups with a similar ANA genetic profile, such as the Xianbei.

The remaining 3 of 18 individuals are excavated from the same mortuary complex, THL-82, and require a distinct eastern Eurasian ancestral component. Two individuals from satellite graves, TAK008 and TAK009, have a high proportion of western Eurasian ancestry but are not modeled as a sister clade with either Chandman_IA or earlyXiongnu_west (qpWave *P* = 1.98 × 10^−7^ for Chandman_IA and qpWave *P* = 2.42 × 10^−3^ for earlyXiongnu_west). We then compared their genetic profile with earlyXiongnu_west by calculating *f*_4_(Mbuti, world-wide; TAK008/TAK009, earlyXiongnu_west) for a set of world-wide ancient and present-day populations. While there is no population showing significant extra affinity with earlyXiongnu_west, several populations show extra affinity with TAK008/TAK009. For TAK009, the top signals are mostly ancient individuals/populations from East Asia (fig. S4). In line with this, we adequately model TAK009 as a mixture of earlyXiongnu_west and various East Asian populations, including Khovsgol_LBA, SlabGrave1, and Han_2000BP (data file S2C). For TAK008, we observe an overall similar trend but find populations with high Ancient North Eurasian (ANE) affinity, such as the Upper Paleolithic individual from the Ust-Kyakhta-3 site (UKY) (*28*) and present-day Native Americans (Mixe and Quechua), among the top signals (fig. S4). Consistent with this, TAK008 is adequately modeled with ~10% contribution from Khovsgol_LBA or UKY, but not with other East Asian proxies with no ANE affinity, such as SlabGrave1 or Han_2000BP (data file S2C). TAK001, a previously published female from a square tomb (*14*), is well explained by the same model with TAK008 and TAK009 but with different admixture proportions. TAK001 derives 90.7% of her ancestry from Khovsgol_LBA and the rest from earlyXiongnu_west. It is rather unexpected to observe the presence of Khovsgol_LBA ancestry in a form not associated with SlabGrave ancestry during the Xiongnu period, as it had largely been replaced by Slab Grave in Mongolia by the EIA (*14*). Khovsgol_LBA ancestry was also reported from the Mongol era site Khalzan Khoshuu, which is located only 95 km away from the TAK site (*14*). Further sampling is required to understand the spatial and temporal distribution of Khovsgol_LBA ancestry after the LBA, especially in the Altai region.

### High genetic diversity within Xiongnu communities and across the empire

**Fig. 3.**
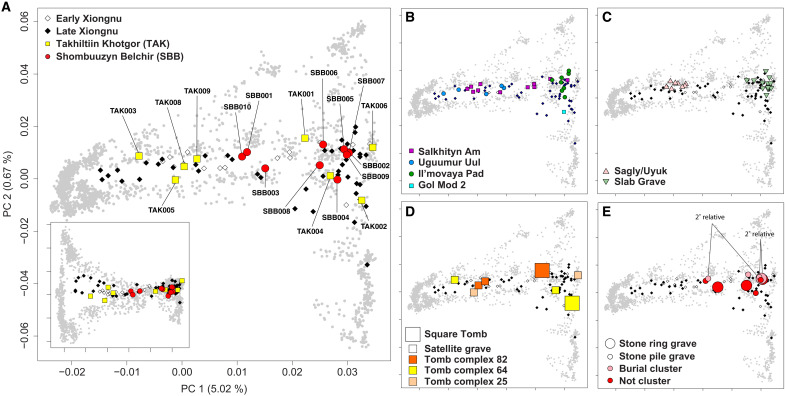
Genetic diversity of the Xiongnu. (**A**) Principal components analysis (PCA) of Takhiltyn Khotgor (TAK) (yellow squares) and Shombuuzyn Belchir (SBB) (red circles) individuals. Other Xiongnu individuals are shown as hollow diamonds (early Xiongnu) and black diamonds (late Xiongnu). Ancient individuals were projected on the PCs calculated with 2077 present-day Eurasian individuals (gray). Inset shows PC1 on the x-axis and PC3 on the *y* axis. PC3 explains the 0.33% of the total variance. The *x* axis and *y* axis ticks have the same values across all panels, except the inset of (A) where the *y* axis ranges from −0.04 to 0.06. (**B**) PCA of Gol Mod 2 (azure squares) and other Xiongnu sites with five or more genetically analyzed individuals: Salkhityn Am (SKT) (purple squares), Uguumur Uul (blue circles), and Il’movaya Pad (IMA) (green circles). (**C**) PCA of the preceding Early Iron Age (EIA) Sagly/Uyuk (pink triangles) and Slab Grave (green down-pointing triangles) individuals. (**D**) PCA of TAK individuals, with color indicating tomb complex and size reflecting grave type. (**E**) PCA of SBB individuals, with color indicating cluster membership and size reflecting grave type. SBB005 is a second-degree genetic relative of SBB001 and SBB007.

**Fig. 4.**
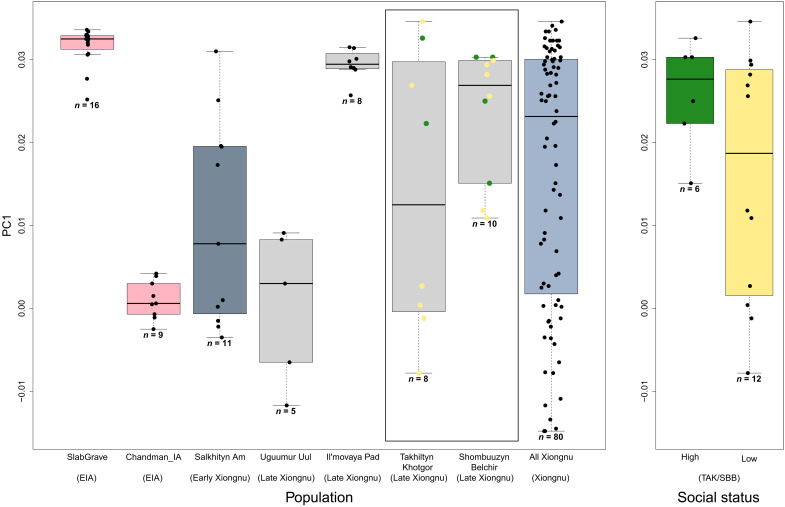
Genetic diversity of the Xiongnu at local and imperial scales. Left presents PC1 coordinates for individuals at Takhiltyn Khotgor (TAK), Shombuuzyn Belchir (SBB), and three previously reported Xiongnu sites with five or more sequenced individuals (*14*). For comparison, we also display PC1 coordinates for Xiongnu, Chandman_IA, and SlabGrave individuals as three independent clusters. The color of the boxes indicates the time period: Early Iron, pink; early Xiongnu, dark gray; late Xiongnu, light gray; and entire Xiongnu, light blue. Newly reported sites (TAK and SBB) are marked by a surrounding box. The PC1 distribution of Xiongnu individuals as a whole exceeds that of the prior Early Iron Age (EIA). With the exception of Il’movaya Pad (IMA), the PC1 distribution of individuals within each Xiongnu site is broader than that of either Chandman_IA or SlabGrave, and the overall PC1 distribution of individuals at Xiongnu sites is generally high. This indicates that the high genetic diversity observed among the Xiongnu as a whole is also reflected within Xiongnu communities. On the right, we compare PC1 coordinates of TAK/SBB individuals according to their social status (high versus low). The color of the boxes indicates social status: green, high; yellow, low; and it matches the color of the dots on the left. The overall PC1 distribution of the high-class individuals is more restricted than that of the lower class individuals.

To examine spatial patterns of Xiongnu genetic diversity at TAK and SBB, as well as across their empire as a whole, we performed principal components analysis (PCA) (Fig. 3) following the approach described by (*66*), projecting ancient individuals onto the genotype dataset of present-day individuals genotyped on the Affymetrix Axiom Genome-Wide Human Origins 1 (“HO”) array (data file S1B) (*59*). All new Xiongnu individuals fall within the diverse range of genetic profiles previously reported for the Xiongnu (*14*) and are widely scattered along PC1 between western and eastern Eurasians. This pattern indicates that the marked genetic heterogeneity observed for the Xiongnu as a whole was also present at sites along its western frontier, far from the imperial core. We next quantified the level of genetic heterogeneity within each site and compared it with the overall Xiongnu genetic diversity within Mongolia (Fig. 4). We used the PC1 coordinates of each individual as a primary variable of the analysis because PC1 captures the major axis of genetic variation within both the Xiongnu and Eurasians in general: High and low PC1 values represent high genetic affinity to eastern and western Eurasians, respectively.

On the basis of the broad distribution of SBB and TAK individuals along the PC1 axis, we find that each site is highly heterogeneous. To statistically compare the genetic diversity of each site and the Xiongnu as a whole (“all Xiongnu”), we applied the Brown-Forsythe test for the null hypothesis that two groups have equal PC1 variances (data file S1D). TAK is highly heterogeneous to the level indistinguishable from the variance across all Xiongnu individuals (*P* = 0.622), while SBB shows less diversity than all Xiongnu (*P* = 0.030). We also compared the genetic diversity of the previously published Xiongnu sites with at least five sampled individuals (*14*). The early Xiongnu site Salkhityn Am (SKT; *n* = 11) in northern Mongolia exhibits a high degree of heterogeneity similar to all Xiongnu (*P* = 0.411). The late Xiongnu site of Uguumur Uul (UGU; *n* = 5) located further to the east shows a more intermediate level of diversity with higher contributions of western Eurasian ancestry, but is still statistically comparable to the all Xiongnu (*P* = 0.185); however, the small sample size for this site provides limited statistical resolution. Still, UGU shows much higher diversity than the preceding EIA groups (*P* = 0.001 and 0.015 for SlabGrave and Chandman_IA, respectively). In contrast, another late Xiongnu site that is located further to the northeast, Il’movaya Pad (IMA; *n* = 8), has predominantly eastern Eurasian ancestry. Its genetic diversity is much lower than all Xiongnu (*P* = 0.002), and is comparable to those of the preceding EIA groups (*P* = 0.975 and 0.398 for SlabGrave and Chandman_IA, respectively) (Fig. 4). We note that the low genetic diversity at the IMA site may be an artifact of biased sampling as only eight individuals were analyzed among 300 circular grave and square tomb burials at the site (*67*). By repeating PC coordinates calculation 100 times for each individual with genotype data downsampled to 10,000 SNPs, we found that the results were not affected by higher uncertainty in PC coordinates in low-coverage individuals (fig. S5).

The comparable level of genetic diversity of all Xiongnu individuals broadly and local communities suggests that the dynamic demographic processes that constituted the highly heterogeneous Xiongnu empire also occurred at local scales. High genetic diversity within the cemeteries of TAK, SBB, and UGU confirm the coresidence of individuals with diverse genetic backgrounds within a single local community and that this continued even into the late Xiongnu period, centuries after the political formation of the Xiongnu and the associated demographic processes of genetic admixture that were already in progress during the initial early Xiongnu period. Overall, the high genetic diversity found among the Xiongnu during all periods prevents any meaningful attempt to define a “representative” Xiongnu genetic profile, as it is instead population-level genetic heterogeneity spanning nearly the entire breadth of Eurasian genetic diversity that most characterizes the Xiongnu Empire.

### Genetic diversity and archaeological signifiers of social status

To better understand how Xiongnu genetic diversity might be structured by social status or social group affiliation, we examined archaeogenetic data from the aristocratic elite cemetery of TAK (Fig. 3D) and the local elite cemetery of SBB (Fig. 3E) separately. At TAK, two complete square tomb complexes have been excavated (THL-82 and THL-64), allowing genetic diversity within discrete mortuary units to be investigated. For the richest tomb complex, THL-82, which contained an older adult female buried in a square tomb (TAK001) flanked by two satellite graves of adult males (TAK008 and TAK009), we identified no genetic relatedness between them. While the genetic profile of the high-status female strongly differed from that of the two low-status males (Fig. 3D), the latter were genetically similar to each other and had much higher levels of western Eurasian ancestry (Fig. 2B and data file S2C). That is, these two males are collectively modeled with 86.8% earlyXiongnu_west ancestry, while TAK001 requires only 9.3% of this ancestry component. In contrast to the high western ancestry of the low-status males, the high-status women (TAK001) shows a high level of eastern ancestry, represented by Khovsgol_LBA, not SlabGrave1.

For the other square tomb complex, THL-64, which contained an adult female buried in a square tomb (TAK002) along with two satellite graves of adolescents, we determined that both adolescents were males. Unlike THL-82, however, the two low-status young males were genetically dissimilar (Fig. 2B and data file S2C), with one (TAK003) having a very high level of western Eurasian ancestry (Chandman_IA and Gonur1_BA) and the other (TAK004) having a high level of eastern Eurasian ancestry (SlabGrave1). The high-status female likewise had a high degree of eastern Eurasian ancestry (Fig. 3D) deriving from two sources (SlabGrave1 and Han_2000BP).

Last, we analyzed the three satellite graves associated with THL-25, for which the square tomb has not been excavated. Two of the three individuals yielded sufficient DNA for analysis: a child (TAK005) and an adult male (TAK006). We determined that both were unrelated males and that they were highly genetically dissimilar (Fig. 3D). The child had a very high level of western Eurasian ancestry (Chandman_IA), while the adult male had the highest eastern Eurasian ancestry (SlabGrave1 and Han_2000BP) observed at the TAK site. Thus, we find very high genetic diversity within individual tomb complexes at the TAK cemetery. Although both high-status females had relatively high levels of eastern Eurasian ancestry, the low-status satellite males exhibited extremely high genetic heterogeneity ranging from very high levels of western Eurasian ancestry to very high levels of eastern Eurasian ancestry. If the low-status males were retainers or servants of the high-status females, it suggests that they were drawn from diverse parts of the Xiongnu empire and possibly beyond.

At the SBB site, we found lower overall genetic diversity, and specifically, no individuals with very high levels of western Eurasian ancestry (Fig. 3E). However, the individuals with the highest levels of western Eurasian ancestry were both adult males (SBB001 and SBB010), although they derived their western ancestry from slightly different sources (Chandman_IA for SBB001 and Chandman_IA and Gonur1_BA for SBB010). As at TAK, the highest-status graves belonged to females (SBB002, SBB003, SBB007, and SBB008), whose modeled ancestries all included SlabGrave1, with other minor ancestry contributions. The genetic determination of SBB007 as female was particularly noteworthy because the grave goods included horse-riding equipment, a gilded iron belt clasp, and a Han-painted lacquer cup, which have been assumed in other contexts to be accouterments associated with male horse-mounted warriors. Similarly, SBB010, an adult male, was buried with a bone tube case containing an iron needle, indicating that sewing implements were not exclusively associated with women. We also determined the genetic sex of three children (SBB004, SBB005, and SBB006) and one adolescent (SBB009) whose sex was uncertain. SBB005 and SBB006 were females, while SBB004 and SBB009 were males. SBB009, an adolescent 11 to 12 years old, was buried with a child-sized bow similar to the bow buried with the adult male SBB001 and the older adolescent SBB011, corroborating accounts of males in Xiongnu society learning to wield bows at a young age (*68*), likely by a young male’s early teens as in the case of SBB009, but likely not in very early childhood, as evidenced by the absence of such equipment in the grave of SBB004, a child 4 to 6 years old.

Examining spatial relationships of burials at SBB, we did not find a significant correlation between spatial proximity and genetic ancestry profiles (Fig. 3E). To represent the similarity of the genetic ancestry profiles of two individuals, we used the Euclidean distance between two points defined in the space of the top two PCs. We could not reject the null hypothesis that the spatial proximity between two burials and genetic distance was unrelated, with a *P* value of 0.146 provided by the Mantel test. Nor could we support a hypothesis that the individuals in the five-grave cluster in SBB (consisting of SBB001, SBB005, SBB006, and SBB007; SBB011 did not produce analyzable genome-wide data) were more similar in their genetic profiles than the others. To determine this, we replaced the geographic distance of each individual pair with 1 for within-cluster pairs and 0 for the others, respectively (Mantel test *P* value = 0.3085). However, we did find that the relatives were placed significantly closer to each other (Mantel test *P* value = 0.0025), based on the two pairs of related individuals buried next to each other: the pair SBB001 and SBB005 and the pair SBB005 and SBB007. These were the only genetically related individuals identified at either SBB or TAK. Although the adult male SBB001 and the female child SBB005 were second-degree relatives, they were genetically dissimilar to one another, with the male having a much higher level of western Eurasian ancestry (Chandman_IA). SBB005 was also a second-degree relative of SBB007, the high-status woman buried with the gilded belt clasp and Han lacquer cup. Both females shared a minor ancestry component modeled as Han_2000BP. Previous investigations of genome-wide genetic relatedness among the Xiongnu have identified 10 other cases of kinship pairs (*14*). Among these, all pairs were buried within the same site or at closely neighboring sites, and most pairs are genetically similar. However, one pair of second-degree maternally related males at the site of Tamiryn Ulaan Khoshuu (TUH001 and TUH002) in north-central Mongolia likewise showed a relatively high degree of genetic dissimilarity (*14*), with one male having substantially more western Eurasian ancestry than the other, although the difference was not as great as between SBB001 and SBB005. Such extended families containing higher degrees of genetic diversity may have been relatively common among the Xiongnu, but denser sampling of cemeteries will be necessary to identify them. Beyond detecting genetic relatedness (*69*, *70*), we also examined runs of homozygosity (ROH) blocks (*71*). We found that SBB005 had long ROH blocks totaling 55.1 centimorgan (cM), with the longest block extending 40.7 cM (fig. S6), suggesting that she is the offspring of a pair of second-degree relatives. Because consanguinity can distort estimates of genetic relatedness, the degree of genetic relatedness between her (SBB005) and SBB001 and SBB007 may be slightly overestimated. Nevertheless, the overall patterns of genetic diversity, heterogeneity, and genetic relatedness at SBB suggest that some local elite families were highly genetically diverse, with marriages occurring between genetically heterogeneous individuals that created complex networks of extended kinship.

### Genetic dynamics of the Xiongnu elite

Together, we observe a high degree of genetic heterogeneity and diversity at the elite sites of TAK and SBB, with the highest genetic heterogeneity observed among the lowest-status individuals. In contrast, we find that the highest-status individuals in this study, the aristocratic and local elite females, tended to be less diverse (*P* = 0.011 for Brown-Forsythe test to compare the PC1 variances of low-status and high-status individuals) with higher levels of eastern Eurasian ancestry. Eastern Eurasian ancestry is represented by the higher PC1 values and the mean of PC1 of the high-status individuals is significantly greater than that of the low-status individuals (*P* = 0.032 for one-sided Welch’s *t* test to test the null hypothesis that PC1 mean of high-status individuals is equal to that of low-status individuals). This suggests that elite status and power was disproportionately concentrated among individuals who traced their ancestry back to the preceding EIA Slab Grave groups. Three of the six elite females, one low-status female child, and one low-status male had minor ancestry contributions consistent with Han_2000BP (*14*, *72*), suggesting that interregional connections to groups in Han Dynasty China may have been greater, and more complex, than previously understood.

Before this study, only one other individual from an elite square tomb had been analyzed in a genome-wide manner: DA39 from Tomb 1 at the imperial elite site of Gol Mod 2 in central-north Mongolia (*13*). This adult male, buried in one of the largest square tomb complexes excavated to date, surrounded by at least 27 satellite burials, and containing rare exotic items such as Roman glass bowls, was likely a chanyu, or ruler of the empire (*73*, *74*). Like the elite women at the western frontier, he also had very high eastern Eurasian ancestry (deriving 39.3 and 51.9% from SlabGrave1 and Han_2000BP, respectively, and the rest from Chandman_IA; data file S2C) and was genetically similar to TAK002 in tomb THL-64 (Fig. 3B). Such patterns of ancestry, stratified by indicators of status and power, provide clues as to the nature of the political formation of the Xiongnu and the relative power dynamics of the empire’s diverse political actors.

Last, the concentration of wealth and elite status specifically among women in these frontier communities warrants further attention. It has been previously noted that Xiongnu-era female graves in peripheral regions of the empire tend to be especially wealthy and high status (*75*, *76*). The elite women’s graves at TAK and SBB conform to fashions befitting higher-ranking persons in Xiongnu society. The association between the social rank and biological sex is statistically significant with *P* value of 0.002 for the Fisher’s exact test. The only females not elaborately buried in wood-plank coffins were children. The prominent status of Xiongnu women at TAK and SBB speaks to the powerful role of women in the empire and their likely prominent place in strategies for expansion and the integration of new realms and territories.

## DISCUSSION

In this genome-wide archaeogenetic study, we find high genetic heterogeneity among late Xiongnu-era individuals at two cemeteries located along the far western frontier of the Xiongnu empire and describe patterns of genetic diversity related to social status. Overall, we find that genetic heterogeneity is highest among lower-status individuals. In particular, the satellite graves surrounding the elite square tombs at TAK show extreme levels of genetic heterogeneity, suggesting that these individuals, who were likely low-ranking retainers, were drawn from diverse parts of the empire. In contrast, the highest-status individuals at the two sites tended to have lower genetic diversity and a high proportion of ancestry deriving from EIA Slab Grave groups, suggesting that these groups may have disproportionately contributed to the ruling elite during the formation of the Xiongnu empire. Nevertheless, the aristocratic elite mortuary pattern at the frontier cemetery of TAK contrasts with that of the much larger square tomb cemeteries located within the core of the empire, where burial complexes like Gol Mod 2 Tomb 1 are flanked by satellite burials of elite circular graves (*10*), whose mortuary treatment and grave inventories indicate that they were of much higher social rank than the retainers at TAK. Thus, the extreme genetic heterogeneity within satellite graves observed at TAK may be more typical of frontier contexts, but further research at core imperial cemeteries is needed to understand these dynamics.

Despite the general trends associating genetic diversity and status, we also identified Xiongnu families at the sites of SBB and TUH whose extended kin networks were genetically diverse, providing a snapshot of the admixture processes that shaped the Xiongnu genetic diversity. Of note, such unions were not limited to the early formative phase of the Xiongnu empire but continued throughout the late Xiongnu period. In contrast to some previous investigations of Xiongnu cemeteries (*12*, *16*), we did not identify close (first-degree) relatives within the cemeteries nor did we observe a strong correlation between grave location and similarity in genetic profiles; however, we did observe two cases of second-degree relatives buried in close proximity to one another at the SBB site. Future studies with more comprehensive sampling of entire cemeteries are necessary to resolve how and where close kin were buried within different mortuary contexts.

Last, our findings also confirm that the highest-status individuals in this study were females, supporting previous observations that Xiongnu women played an especially prominent role in the expansion and integration of new territories along the empire’s frontier. Building on this work, future research focusing on dense sampling and genome-wide archaeogenetic analysis at large, extensively excavated Xiongnu cemeteries across the empire promises to reveal in high resolution the complex structure of Xiongnu society from its core to its vast frontier.

## MATERIALS AND METHODS

### Archaeological sites and sample description

#### TAK

This cemetery lies along the foothills of the Southern Altai and is a mixture of 34 exclusive square tombs of the Xiongnu aristocracy, 35 small satellite burials flanking these tombs, and 60 large standard elite graves (47.403058N, 92.104914E; 1470-m elevation). Two large square tombs were excavated in the 1990s (*19*), and one of those individuals was later sampled for genetic analysis (TAK001) (*14*). In 2007, the two satellite burials beside that tomb, three satellite burials flanking another tomb, and the entire complex of a third tomb with two satellite burials were excavated, to investigate the differences between aristocratic tombs in the frontier and the core realms and to study the relationships between individuals in square tombs and those who accompanied them in death in the satellite burials (*17*). While the contents and forms of the square tombs correspond as well to those of royal square tombs in central Mongolia, the satellite burials are far smaller and more meager than standard Xiongnu graves and many show burial traditions more akin to pre-Xiongnu local practices than to Xiongnu customs (*10*). Further information about the TAK burials are provided in data file S1A.

#### SBB

This small cemetery of 33 standard local elite graves (circular graves) is located at the far western edge of the Xiongnu realms in the high mountain passes of the Southern Altai (46.921828N, 91.934913E; 2390-m elevation). Fifteen graves were excavated at the site, sampling across the burial ground but also giving special attention to the main cluster, which included males and females as well as a full spectrum of older and younger adults, and children and infant subadults (*20*). The manner of burial goods and funerary practices of these graves correspond to Xiongnu traditions in the core imperial realms, demonstrating a community of local elites who participated fully in the imperial network (*10*). Further information about the SBB burials are provided in data file S1A.

## Sample provenance

Excavations were conducted in 2007, 2008, and 2010 under a permit of the Mongol-American Khovd Archaeology Project granted through the National Museum of Mongolia. The principal investigators of the project were J. Bayarsaikhan (National Museum of Mongolia) and B. K. Miller (at that time, University of Pennsylvania). The burial sites from which all human remains were excavated are (i) Takhiltyn-Khotgor (Mankhan sum, Khovd aimag, Mongolia) and (i) Shombuuzyn-Belchir (Mönkhkhairkhan sum, Khovd aimag, Mongolia). The time period of the burial sites was validated by radiocarbon dating (*18*). The risk of damage or looting of the sites is minimal (3, on scale of 1 to 10). Bone samples used for the genetic analysis were exported from Mongolia to Germany (Max Planck Institute for the Science of Human History, Jena) for analyses under contract no. 2015.03.30, reference no. MN DE 7 14583. All human remains are housed at the National Museum of Mongolia in Ulaanbaatar, Mongolia.

## Sampling for ancient DNA recovery and sequencing

We extracted genomic DNA and prepared single-stranded DNA sequencing library for 8 individuals from TAK site and 11 individuals from SBB site. For this, we followed published protocols (*77*, *78*) in a dedicated ancient clean room facility at the Max Planck Institute for the Science of Human History. During the library preparation, we added unique 8-mer index sequences at both P5 and P7 Illumina adapters for double indexing. Details of the laboratory protocols are available online (https://protocols.io/view/a-z-of-ancient-dna-protocols-for-shotgun-illumina-36wgq529xgk5/v2). We then performed shallow shotgun sequencing to screen 19 individuals and found that 17 individuals are sufficiently preserved with ≥0.1% reads mapped on hs37d5, the human reference genome GRCh37 with decoy sequences. To enrich these libraries for 1,233,013 nuclear SNPs (1240K), we used oligonucleotide probes targeting 1240K sites and performed in-solution DNA capture (*22*). We then generated single-end 76–base pair (bp) sequences on the Illumina HiSeq 4000 platform. For the 6 of 19 individuals with sufficiently high preservation (>30% human DNA), we produced in-depth whole-genome shotgun sequencing data at the Bauer Core Facility of Harvard University. For these shotgun libraries, we generated paired-end 100-bp sequences on Illumina NovaSeq 6000 platform. We demultiplexed the output sequences allowing at most one mismatch in each index. The sequencing scheme of each individual is described in data file S1F.

## Quantification and statistical analysis

### DNA sequence data processing

We trimmed the Illumina adapter sequences at the ends of each read and discarded reads shorter than 35 bp using AdapterRemoval v2.3.0 (*79*). Then, we mapped the retained reads to hs37d5 with the aln and samse modules in the Burrows-Wheeler Aligner program v0.7.17 with noncustom options for disabling seeding (“-l 9999”) and allowing additional mismatches (“-n 0.01”) (*80*). We removed polymerase chain reaction duplicates with dedup v0.12.5 (*81*) and then removed reads with the Phred-scaled mapping quality score lower than 30 using SAMtools v1.9 (*82*). We report the summary statistics based on the 1240K sites in data file S1A.

### Data quality control

Before population genetic analysis, we assessed the authenticity of our ancient DNA sequence data using multiple measures. We first tabulated the postmortem damage pattern of each library using mapDamage v2.0.9 (*83*) and checked whether each library showed increased C-to-T misincorporation at both ends of the reads as expected for single-stranded libraries (data file S1A). We then estimated the mitochondrial contamination rate of all individuals using schmutzi v1.5.4 (*23*). Schmutzi distinguishes between endogenous and contaminant reads based on the deamination frequency and the read lengths. For males, we additionally estimated the nuclear contamination rate using the contamination module of the ANGSD v0.929 program based on the X chromosome data (*24*). In brief, the method compares mismatch rates between multiple reads of known polymorphic sites on X chromosomes and that of flanking monomorphic sites, interpreting higher mismatch rate of polymorphic sites as evidence of contamination because males have only one X chromosome and thus reads from polymorphic sites should not show any more mismatches than those from monomorphic ones.

### Genotyping and dataset compilation

For genotype calling, we randomly chose one high-quality base (Phred-scaled base quality score 30 or higher) from one high-quality read per each position and considered the chosen base as a homozygous genotype for that position (“pseudo-haploid genotype”), using the pileupCaller v1.4.0.5 program with the “randomHaploid” option (https://github.com/stschiff/sequenceTools; v1.5.2 last accessed at 25 April 2022). Because our libraries were single-stranded, we used the “singleStrandMode” option to use only negative strand reads to genotype C/T SNPs and only positive strand reads to genotype G/A SNPs. We then merged genotyped data of 17 new individuals with genotype data of previously published worldwide present-day (*25*, *29*, *59*–*63*) and ancient (*13*–*15*, *22*, *25*–*58*) individuals, typed on the two sets of SNPs: the Affymetrix Axiom Genome-Wide Human Origins 1 array (“HumanOrigins”) (*59*) and a set of 1,233,013 SNPs including the HumanOrigins SNPs (1240K) (data file S1B).

### Sex determination and uniparental haplogroup assignment

To determine the genetic sex of each individual, we estimated the ratio of the sequence coverage of sex chromosomes to that of autosomes. It is expected that the ratio of X to autosomal coverage is ~0.5 for males and ~1 for females and the ratio of Y to autosomal coverage is ~0.5 for males and ~0 for females. The individuals with the ratio of Y to autosomal coverage >0.3 are reported as a male and individuals with the ratio <0.1 are reported as a female (data file S1A). We also retrieved uniparental haplogroups of each individual. First, we generated mitochondrial consensus sequences of quality ≧ 10, using the log2fasta program in the Schmutzi package (*23*). Each sequence is assigned into the specific haplogroup using HaploGrep v2.1.20 (data file S1A) (*84*). For the 10 males, we called 13,508 Y chromosome SNPs from the ISOGG database with pileupCaller v1.4.0.5 option “majorityCall” and assigned Y-chromosomal haplogroup using a modified version of the yHaplo program (*85*) (https://github.com/alexhbnr/yhaplo; version 2016.01.08. last accessed at 28 April 2022) (data file S1A).

### Estimation of genetic relatedness

We calculated the pairwise mismatch rate (pmr) of pseudo-haploid genotypes between every pair of individuals by dividing the number of sites genotyped differently from one another by the number of sites covered by both individuals (*70*). The low pmr value suggests close genetic relatedness between individuals, and we used pmr between unrelated individuals as the baseline to estimate the kinship coefficient. We observed two pairs of genetic relatives with sufficient coverage, SBB001 and SBB005 and SBB005 and SBB007 (data file S1E). SBB005 is inferred to be inbred between second-degree relatives by hapROH (fig. S6) (*71*). HapROH identifies ROH, regions that lack heterozygous genotypes and estimates the degree of inbreeding. Long ROH blocks suggest that parents of the individual are genetically related to each other.

## Population genetic analysis

### Analysis of population structure and relationships

We performed PCA on the merged Human Origins dataset using the smartpca v16000 program in the Eigensoft v7.2.1 package (Fig. 3 and fig. S3) (*66*). With the option “lsqproject: YES,” we first calculated eigenvectors with 2077 present-day Eurasian individuals and projected the ancient individuals on the calculated eigenvectors (*25*). PC1 separates the eastern and western Eurasians and we used PC1 to measure the genetic diversity of the Xiongnu communities (Fig. 4). We also calculated *f*_4_ statistics with option “f4mode: YES” to further understand the genetic profiles of ancient individuals. We used qpDstat v970 from admixtools v7.0 (*59*) on the 1240K dataset to maximize SNP coverage of ancient individuals. SEs were calculated with 5 cM block jackknifing as implemented in the admixtools package (fig. S4).

### Admixture modeling using qpAdm

We modeled ancient individuals as a mixture of source populations and estimated ancestry proportions using the qpWave v1200 and the qpAdm v1201 programs in the admixtools v7.0 package (*25*). We used the 1240K dataset for these analyses and used the following set of 10 populations as the base outgroup set (“right populations”): central African rainforest hunter-gatherer (Mbuti, *n* = 5), Andamanese islander (Onge, *n* = 2), Taiwanese Aborigine (Ami, *n* = 2), Central American (Mixe, *n* = 3), Epipaleolithic Levantine (Natufian, *n* = 6) (*25*), Neolithic Iranians from the Ganj Dareh archaeological site (Iran_N, *n* = 8) (*25*, *26*), Epipaleolithic European hunter-gatherer (Villabruna, *n* = 1) (*33*), Anatolian Neolithic from the Barcin site (Anatolia_N, *n* = 23) (*22*), Eneolithic hunter-gatherer from northern Kazakhstan (Botai_pub, *n* = 3) (*31*), and Neolithic individuals from southern Russia (West_Siberia_N, *n* = 3) (*26*). For Xiongnu individuals, we added Khovsgol_LBA (*n* = 14) (*69*) to the base outgroup set to increase the resolution and to better distinguish the eastern ancestries. When there are multiple combinations of source populations that fit the given target population, we tried to increase the resolution by taking the “rotating” approach (*86*), in which we added source populations in one model to the right populations when testing a competing source combination.

### Statistical tests for sociopolitical and spatiotemporal distribution of the genetic profiles

We performed several tests using the program R v4.1.2 (*87*) to statistically compare the genetic profiles or prove the association between genetic profiles and other factors. To represent the genetic profiles, we used PC values calculated by PCA of present-day Eurasian individuals. We first grouped individuals by the archaeological site or by the social status. We tested the equality of the PC1 mean and variances of the groups using Welch’s *t* test and Brown-Forsythe test, respectively, to compare the genetic ancestry and diversity among groups. For Welch’s *t* test, we used the function t.test of R package stats (v4.1.2; the package “stats” is part of R) and for the Brown-Forsythe test, we used the function leveneTest of R package car (v3.1.0). We also tested the association between the similarity of the genetic profiles and the spatial proximity of the burials. We performed the Mantel test on matrices of physical distances and the Euclidean distances between two points on the PC plane defined by the top two PCs, using the function mantel.rtest of R package ade4 (v1.7.19). To statistically test the association between sex and the social status and explain the sex bias in social status, we performed the Fisher’s exact test using the fisher.test function of R package stats (v4.1.2; the package stats is part of R).

## Notes

View/request a protocol for this paper from *Bio-protocol*.
