# Supplementary material for: Genetic population structure of the Xiongnu Empire at imperial and local scales
Source: Sci Adv. 2023 Apr 14;9(15):eadf3904. doi: 10.1126/sciadv.adf3904 (PMC10104459; doi:10.1126/sciadv.adf3904)
Supplement: Supplementary file 1 — Figs. S1 to S6 Legends for data S1 and S2 [file sciadv.adf3904_sm.pdf]

Science Advances
AAAS

Supplementary Materials for

Genetic population structure of the Xiongnu Empire at imperial and local scales

Juhyeon Lee *et al.*

Corresponding author: Choongwon Jeong, cwjeong@snu.ac.kr; Christina Warinner, warinner@fas.harvard.edu; Bryan K. Miller, bkmil@umich.edu

Sci. Adv. **9**, eadf3904 (2023)
DOI: 10.1126/sciadv.adf3904

The PDF file includes:

Figs. S1 to S6
Legends for data S1 and S2

Other Supplementary Material for this manuscript includes the following:

Data S1 and S2

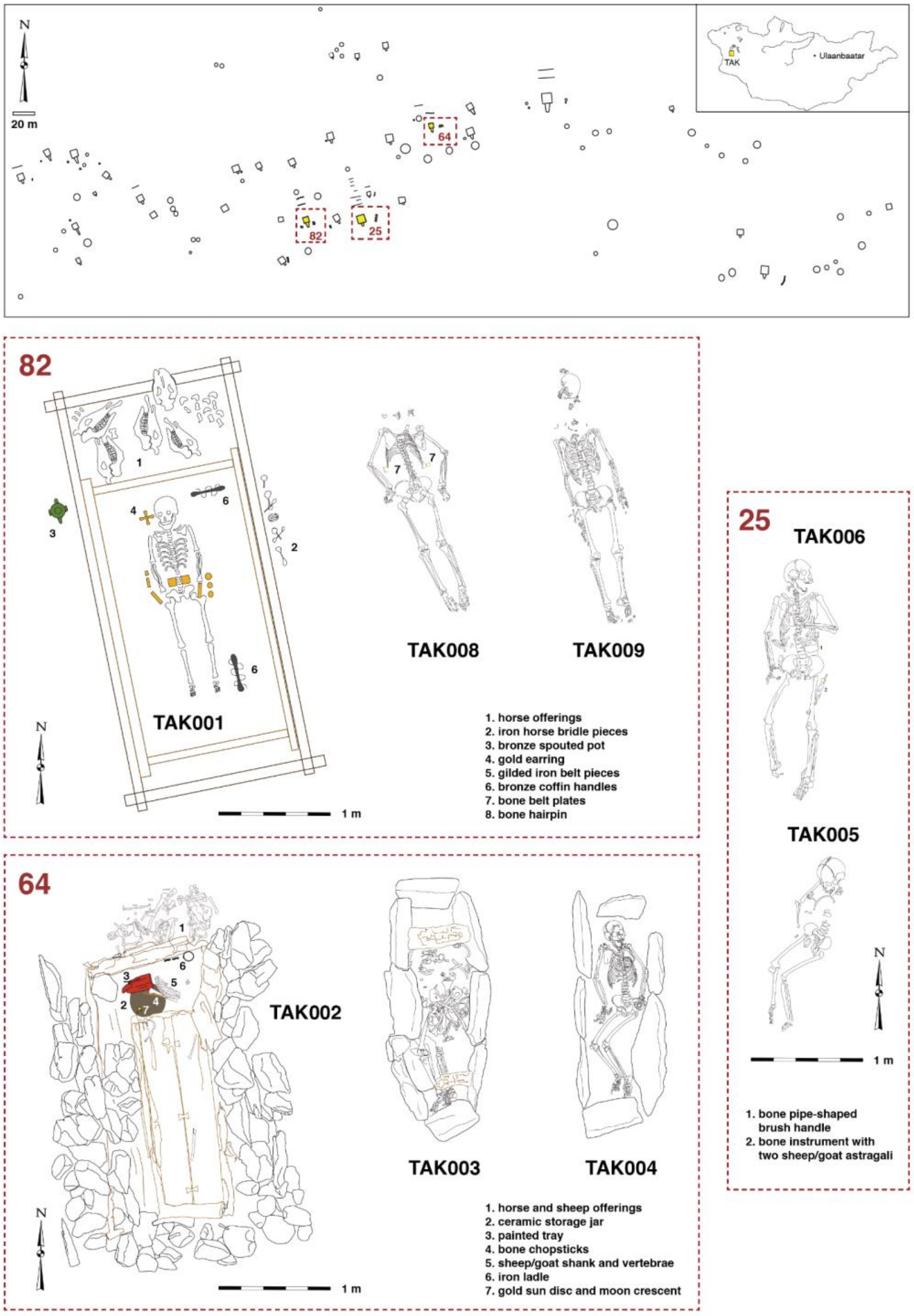

Fig. S1. Plan of the imperial elite cemetery at Takhiltyn Khotgor and burial details of the square tomb complexes analyzed in this study.

Tomb complexes 82, 64, and 25 are marked with dashed red boxes in the map, and are shown below in more detail. Burial drawings are to scale and the shape (squares or circles) reflects the burial type. Further information about each burial, including mortuary characteristics, osteological information, and associated grave good inventories, are provided in Data S1A.

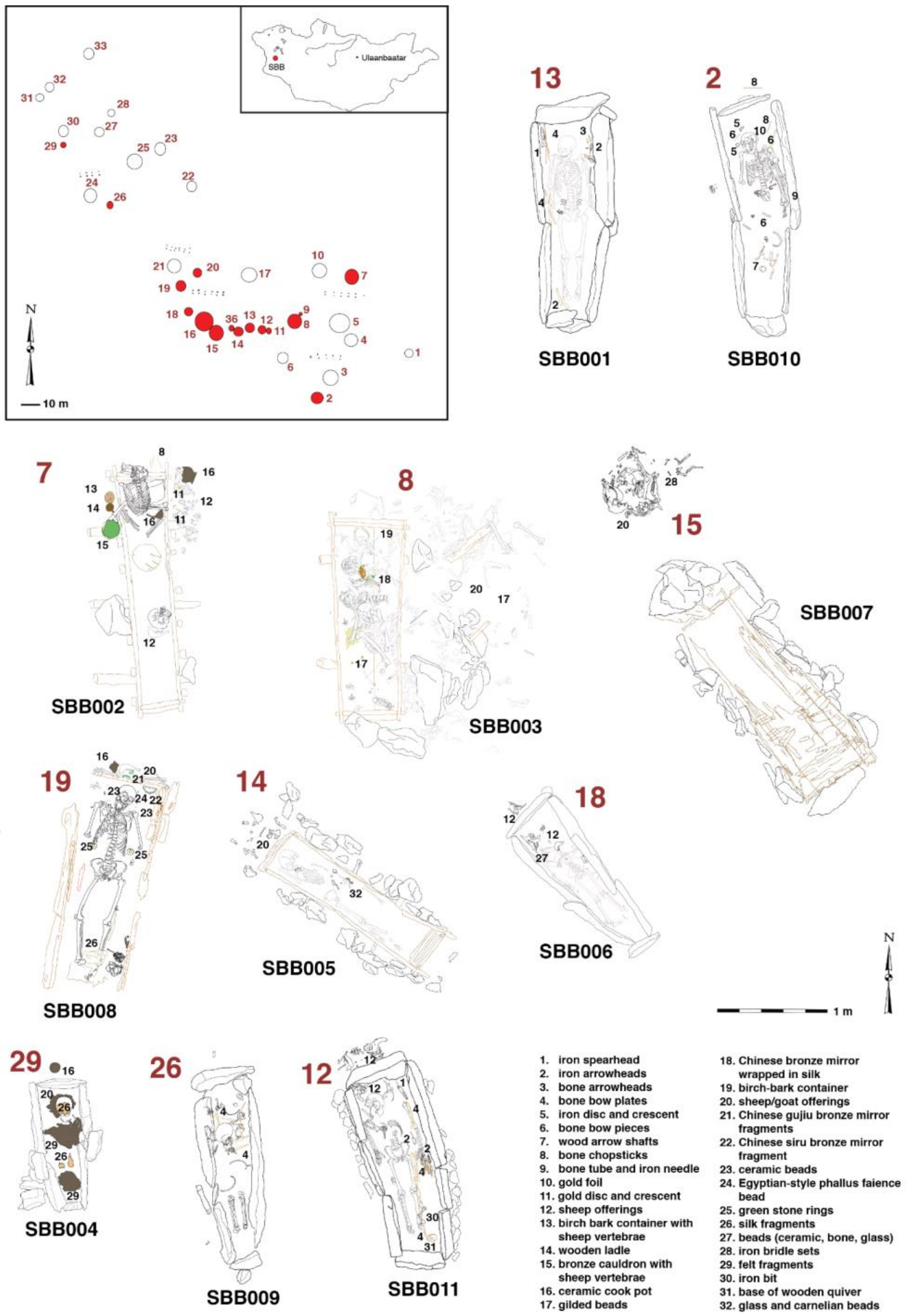

Fig. S2. Plan of the local elite cemetery at Shombuuzyn Belchir (SBB) and burial details of the graves analyzed in this study.

Analyzed graves are marked in red, and are shown below in more detail. Burial drawings are to scale. Further information about each burial, including mortuary characteristics, osteological information, and associated grave good inventories, are provided in Data S1A. Note that grave goods recovered from a different stratigraphic level than the coffin (e.g., due to looting or other displacement) are not shown in the burial drawings, but they are described in Data S1A. Traces of perishable items (e.g., silk, wool) are indicated in the drawings only if significantly preserved.

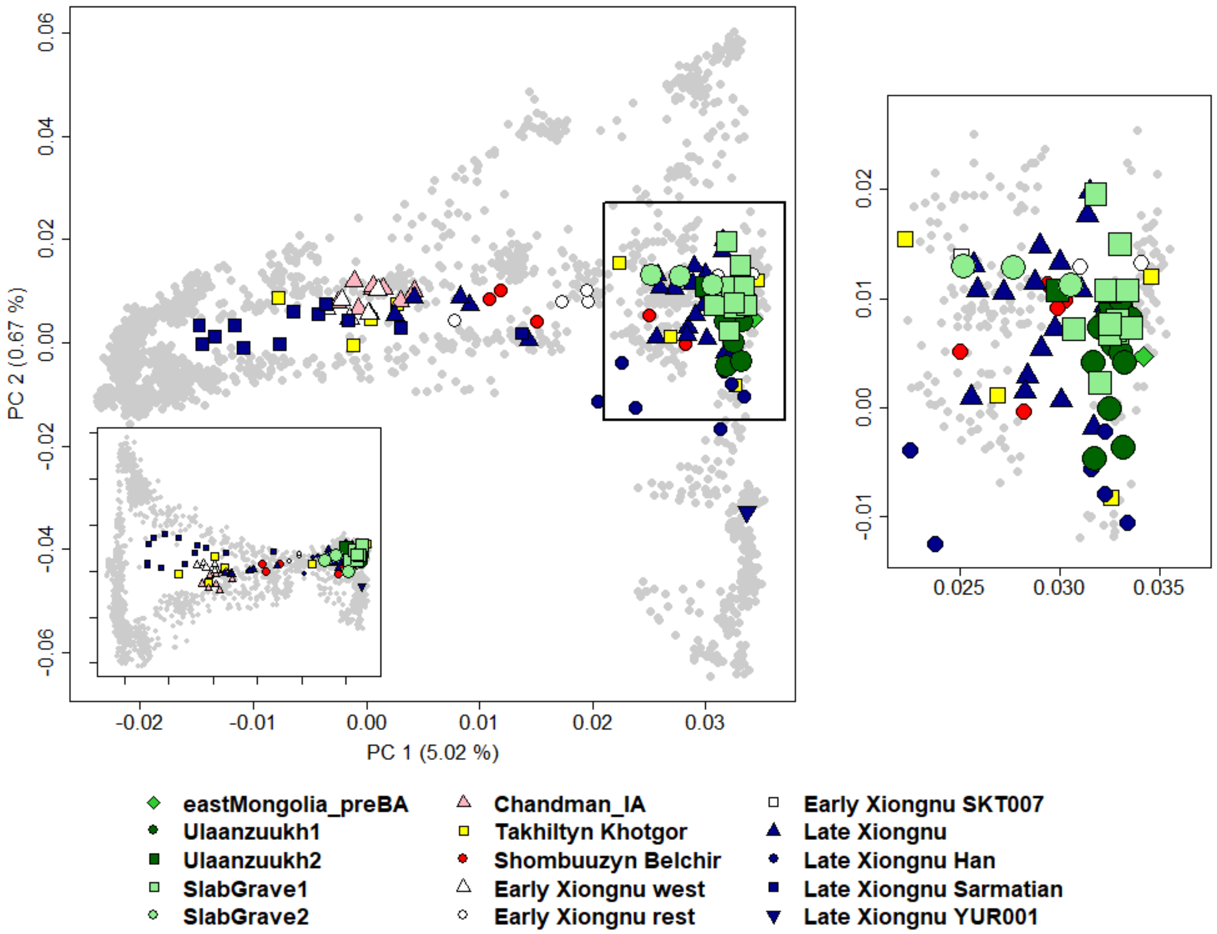

Fig. S3. Principal Component Analysis of Ulaanzuukh and Slab Grave individuals.

The ancient samples (color-filled shapes) were projected onto the PCs calculated by 2,077 modern Eurasians (marked in small grey circles). Ulaanzuukh and SlabGrave individuals are highlighted with bigger symbols. Main panel on the left plots PC1 to PC2 and the inset panel plots PC1 to PC3. PC3 explains the 0.33% of the total variance. The ticks for the x axis of the inset panel are equal to that of the main panel and y-axis ranges from -0.04 to 0.06 by the increment of 0.02. The zoom-in figure of the region surrounded by the black box is shown on the right. The colors and shapes indicate the population analysis unit used in the study and the colors match to Figure 3 in the main text.

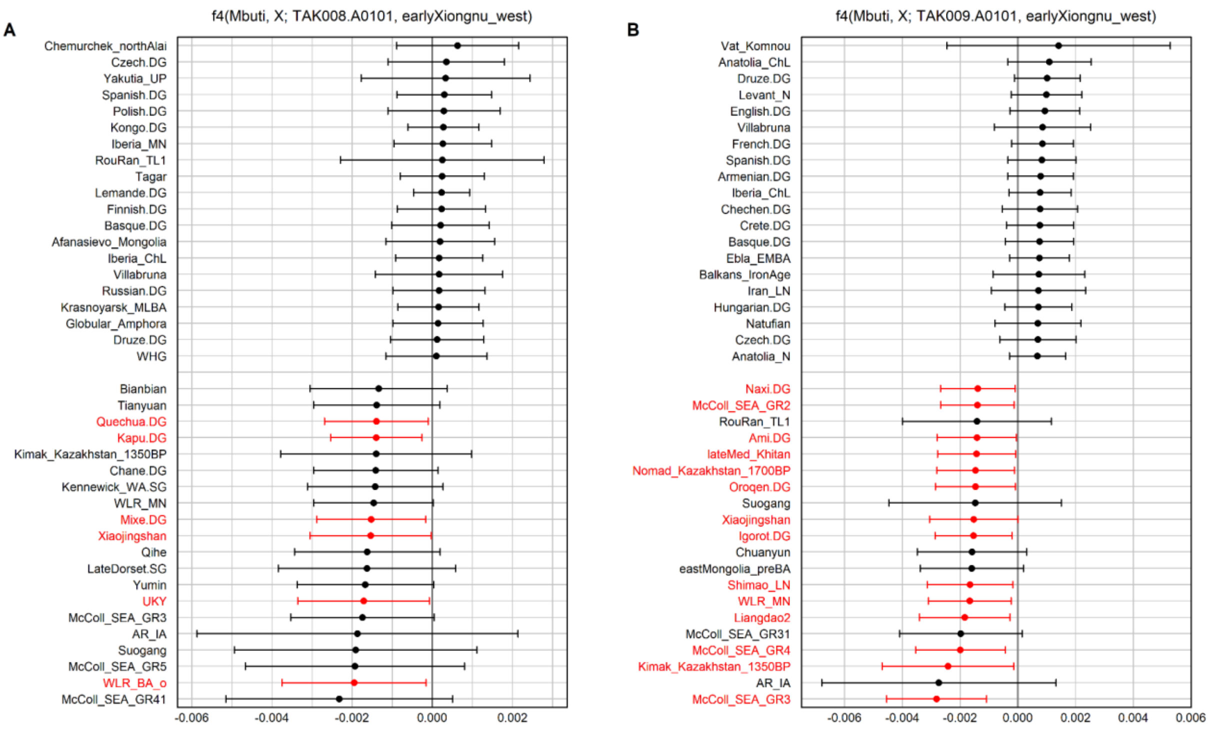

Fig. S4. f4 statistics to test the cladality of TAK008/TAK009 and earlyXiongnu_west. We compare genetic profiles of TAK008/TAK009 and earlyXiongnu_west by calculating f4(Mbuti, world-wide; TAK008/TAK009, earlyXiongnu_west). We present the results of top and bottom 20 populations with more than 20,000 SNPs used. The circle indicates f_4 value and the horizontal line indicates ±3 standard errors estimated by 5 cM block jackknifing. The results with | Z | > 3 are shown in red. (A) Results of f_4(Mbuti, world-wide; TAK008, earlyXiongnu_west) showing that TAK008 shares extra affinity with populations with Ancient North Eurasian ancestry. (B) Results of f_4(Mbuti, world-wide; TAK009, earlyXiongnu_west) showing that TAK009 shares extra affinity with ancient populations from East Asia.

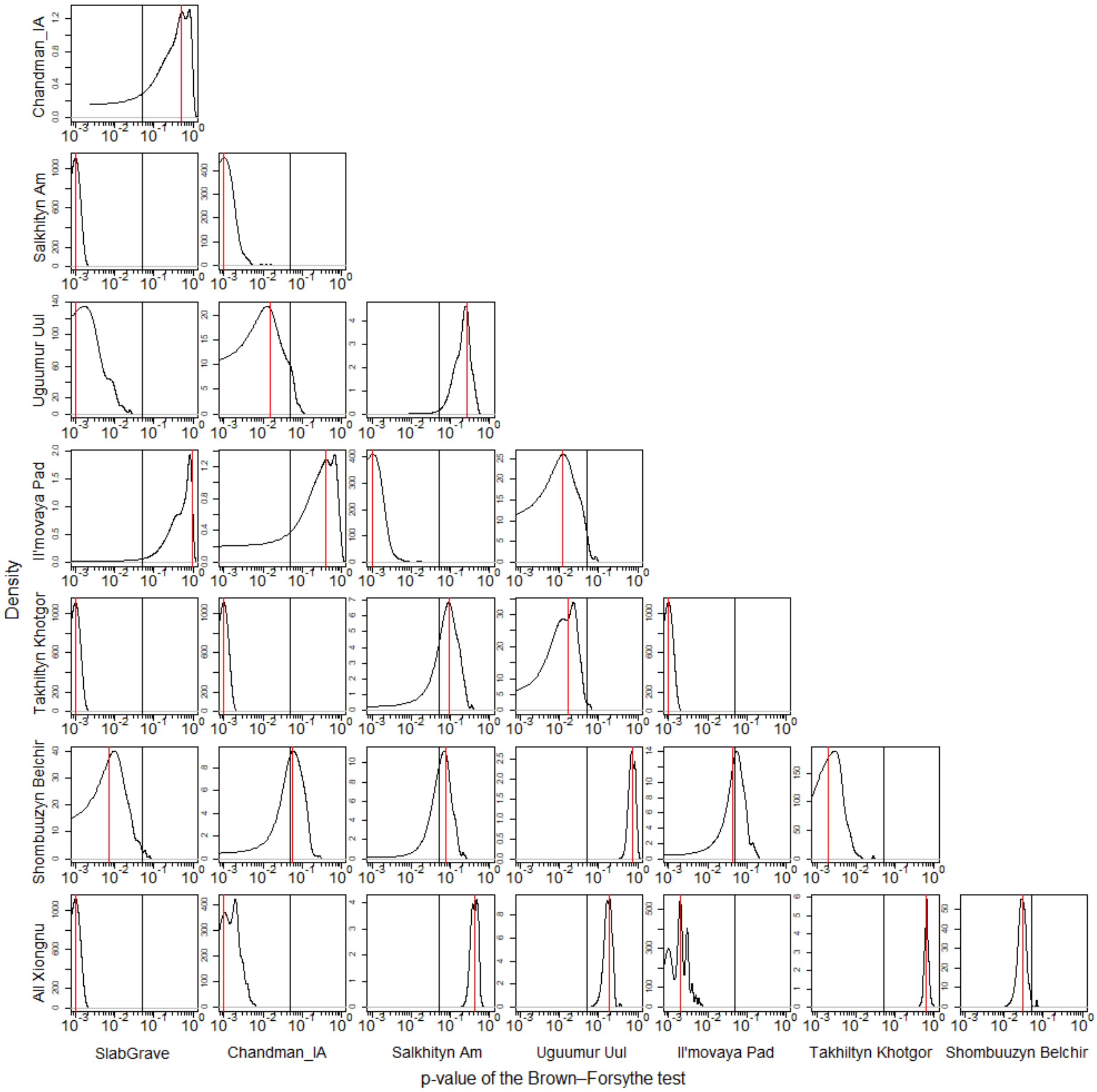

Fig. S5. SNP down-sampling results for the Brown-Forsythe test of the genetic heterogeneity between different Xiongnu sites.

Each cell shows a comparison between the two groups labeled on the x- and y-axis, respectively. Black curve shows the distribution of 100 p-values obtained from repeated down-sampling experiments. The red vertical line shows the p-value with all data without down-sampling. P-values less than 0.001 are shown as 0.001. The black vertical line shows p=0.05. P-values from the down-sampling replicates are tightly distributed around the all data p-value.

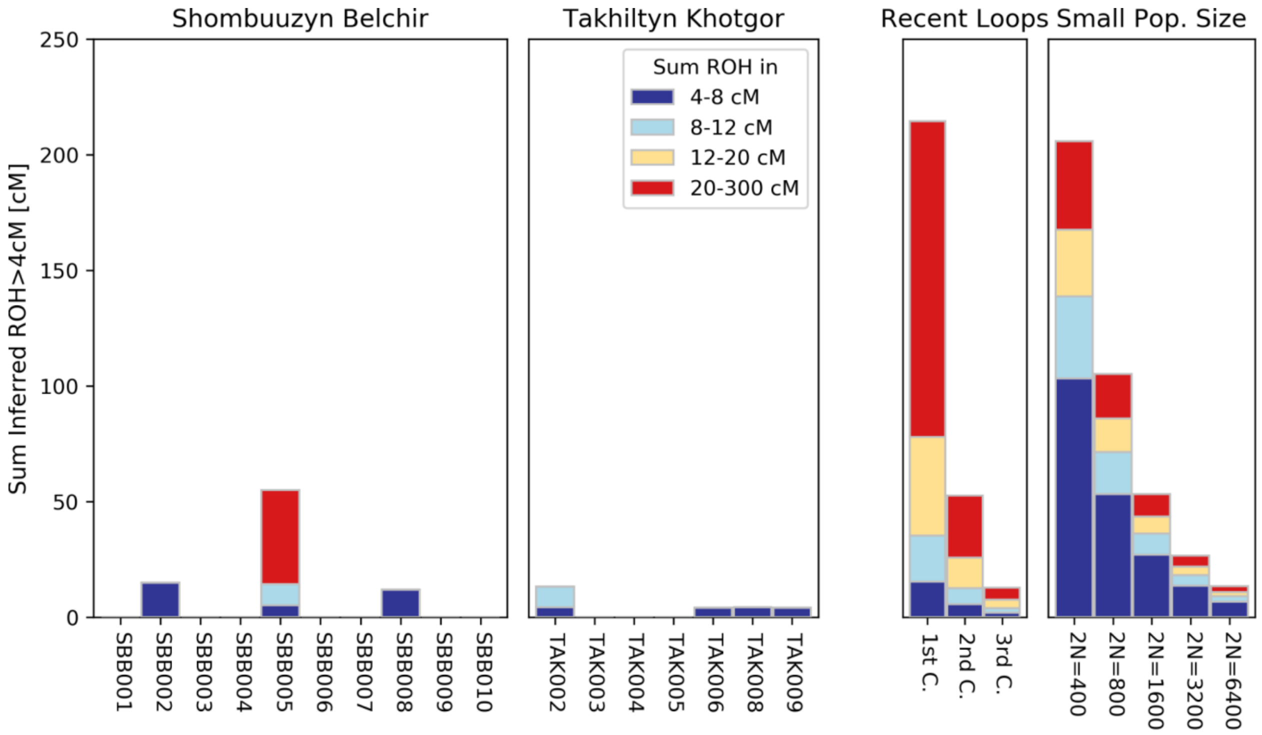

Fig. S6. HapROH results of TAK/SBB individuals.

We present the length of Runs of Homozygosity (ROH) blocks for each individual. The ROH blocks are sorted by their length, which is indicated by colors. The expected distributions of ROH blocks for recent consanguinity and small population size are shown on the right. SBB005 is expected to be inbred between second-degree relatives and others show no signal of recent inbreeding.

Supplementary Data files

The following data files are provided as separate spreadsheet files:

Data S1.
Information about the newly reported ancient individuals, the list of ancient and modern populations used in this study, the list of Y haplogroups of ancient Eastern Steppe individuals, the table for the p-values of the Brown–Forsythe test, the list of genetic relatives newly reported in this study, and the table for the sequencing scheme.

Data S2.
Results of all the qpWave/qpAdm analyses reported in this study.
